# Ovarian preservation in adenocarcinoma of the uterine cervix

**DOI:** 10.1186/s13048-017-0339-y

**Published:** 2017-07-24

**Authors:** Jiansong Zhou, Yuanyuan Chen, Ping Zhang, Hanmei Lou

**Affiliations:** 1 The Key Laboratory of Radiation Oncology of Zhejiang Province, Department of Gynecologic Radiation Oncology, Zhejiang Cancer Hospital, Hangzhou, Zhejiang People’s Republic of China; 2 Department of Radiation Oncology, Hangzhou Cancer Hospital, Hangzhou, Zhejiang People’s Republic of China; 3 Department of Gynecologic Oncology, Zhejiang Cancer Hospital, Hangzhou, Zhejiang People’s Republic of China

**Keywords:** Adenocarcinoma, Ovarian metastasis, Ovarian preservation

## Abstract

**Background:**

An upward trending incidence in cervical adenocarcinoma (ADC) has been reported in many countries. Because non-squamous histology has been associated with increased risk of ovarian metastases (OM), bilateral oophorectomy is commonly performed for ADC without due consideration for ovarian preservation, degrading the quality of life for young premenopausal patients.

**Methods:**

Subjects were patients with International Federation of Gynecology and Obstetrics (FIGO) stage I–IIB cervical ADC who underwent radical hysterectomy, including pelvic lymphadenectomy and bilateral salpingo-oophorectomy at our institution between Oct. 2006 and Sept. 2014. Clinicopathologic variables were studied by univariate and multivariate analyses.

**Results:**

Of the 312 patients enrolled in the study, 14 patients (4.5%) developed OM. Multivariate analysis revealed that uterine corpus involvement (odds ratio [OR] 5.178, *p* = 0.019), parametrial involvement (OR 14.125, *p* = 0.005) and vaginal infiltration (OR 4.167, *p* = 0.047) were independently associated with metastasis. OM had no effect on either relapse-free survival (95% confidence interval [CI]: 0.077–4.095, *p* = 0.57) or overall survival (95% CI: 0.893–9.820, *p* = 0.076).

**Conclusion:**

Cervical ADC is associated with an increased risk of OM. Ovarian preservation surgery in cervical ADC may be safe for young patients at an early FIGO stage without deep stromal, endometrial or perineural invasion, and particularly without uterine corpus invasion, parametrial involvement and infiltration into the vagina.

## Background

Invasive cervical cancer (ICC) ranks as the third most common malignancy and is the fourth leading cause of female cancer deaths worldwide [1]. Patients with FIGO early stage disease generally undergo radical hysterectomy and pelvic lymphadenectomy. However, bilateral oophorectomy is not part of standard surgical management of ICC.

Currently, an upward trend in the incidence of adenocarcinoma (ADC) has been reported in many countries, particularly among women under the age of 40 [2–5]. Since Shimada et al. and Ronnett et al. have reported that approximately 5% of women with cervical ADC are at an increased risk of ovarian metastases (OM), which occurs in about half of ADC cases post-hysterectomy [6, 7], oophorectomy is commonly performed in ADC to preclude OM.

Ovarian preservation, which is beneficial to the physiologic and psychosexual well-being of premenopausal women affected by cervical cancer, remains a challenge in clinical practice. Some believe that non-squamous histology should be a deterrent to ovarian transposition [8]. Others advocate lymphovascular space invasion (LVSI), deep stromal invasion (DSI) and uterine corpus involvement as contraindications [8, 9]. The aim of the present retrospective study was to identify the clinicopathological factors associated with OM in ADC.

## Methods

### Patients

Study participants were patients diagnosed with FIGO stage I–IIB invasive ADC of the uterine cervix and treated by radical hysterectomy, bilateral salpingo-oophorectomy and pelvic lymphadenectomy at the Zhejiang Cancer Hospital, Zhejiang Province, China between Oct. 2006 and Sep. 2014. Clinical data were extracted from the institution’s electronic databases after informed consent was obtained from all patients. The Medical Ethics Committee of Zhejiang Cancer Hospital approved the study.

The following clinical and histological parameters were evaluated in relation to OM: age at surgery, FIGO stage, bulky tumor size (>4 cm), differentiation, morphology, DSI (≥2/3), LVSI, parametrial involvement, uterine corpus involvement, endometrial invasion, fallopian tube invasion, vaginal infiltration, perineural invasion (PNI) and para-aortic/pelvic lymph node status.

### Statistical analysis

Statistical analyses were performed with the SPSS 16.0 software package (IBM, Armonk, NY, USA). *P*-values <0.05 were considered statistically significant. Summary statistics are presented as frequencies and percentages. The Pearson χ^2^ test was used to assess the association between clinicopathologic parameters and the presence of OM. Multivariate analysis was used to detect independent risk factors for OM and results are presented as odds ratios (OR).

## Results

### Patient characteristics

A total of 312 ADC patients were enrolled into the study, including 9 patients (2.9%) with FIGO stage IA, 217 patients (69.6%) with stage IB, 74 patients (23.7%) with stage IIA and 12 patients (3.8%) with stage IIB. The median age of patients was 46 years (range: 19–73 years).

OM were diagnosed in 14 patients (4.5%). A summary of patients with OM is shown in Table 1. OM occurred in five of the 217 patients (2.3%) in stage IB, eight of the 74 patients (10.8%) in stage IIA and one of the 12 patients (8.3%) in stage IIB. A significantly higher incidence of OM was observed in stage II ADC (*p* = 0.002, OR 9.899). The mean age of these 14 patients was 46 (range 32–68) years. Potential risk factors for OM are listed in Table 2. Patients with OM were frequently observed by univariate analysis with FIGO stage I or II (*p* = 0.002), DSI (*p* = 0.002), uterine corpus involvement (*p* < 0.001), endometrial invasion (*p* < 0.001), parametrial involvement (*p* < 0.001), PNI (*p* = 0.011), fallopian tube invasion (*p* < 0.001) or vaginal infiltration (*p* < 0.001). However, outcomes for patients with OM did not correlate with age at surgery, bulky tumor size, differentiation, morphology, LVSI or lymph node metastasis.

**Table 1:** Summary of patients with ovarian metastasis

Case	Age	FIGO stage	Grade	DSI	Morphology	UCI	Endometrial invasion	Fallopian tube invasion	Perineural invasion	LVSI	Infiltration to vagina	Lymph node metastasis	
												Para-aortic	Pelvic
1	42	IIA	G3	+	Endophytic	+	−	−	+	+	−	+	+
2	43	IIA	G2	+	Exophytic	−	−	−	+	−	+	−	−
3	62	IIA	G2	+	Endophytic	+	−	−	+	−	+	−	+
4	49	IB	G3	+	Exophytic	+	−	+	−	+	+	−	−
5	41	IIA	G2	+	Exophytic	−	−	−	−	+	+	−	+
6	51	IIB	G2	+	Exophytic	−	−	−	−	−	−	−	−
7	36	IIA	G3	−	Exophytic	+	+	−	−	−	−	−	−
8	55	IIA	G2		Exophytic	−	−	−	−	−	+	−	+
9	48	IIA	G2		Endophytic	−	−	−	+	+	+	−	−
10	36	IB	G3	−	Exophytic	+	−	−	−	−	−	−	−
11	43	IB	G2	−	Exophytic	+	−	−	−	−	+	−	−
12	68	IB	G3	−	Endophytic	+	+	+	−	+	+	−	−
13	43	IIA	G2	+	Endophytic	+	+	+	+	+	−	−	−
14	32	IB	G3	+	Endophytic	+	−	+	−	+	−	−	−

*DSI* deep stromal invasion

*UCI* uterine corpus involvement

*LVSI* lymphovascular space invasion

**Table 2:** Univariate and multivariate analyses of risk factors for ovarian metastasis in patients with cervical adenocarcinoma

Parameter	N (%)	Univariate analyses			Multivariate analyses	
Total no. of patients enrolled	312	Ovarian metastasis			Odds ratio	*p*-value
		Yes	No	*p*-value		
Age at surgery						
< 60	282 (90.4)	12	270	0.544		
≥ 60	30 (9.6)	2	28			
FIGO stage						
I	226 (72.4)	5	221	0.002*	2.445	0.243
II	86 (27.6)	9	77			
Tumor diameter (cm)						
< 4	221 (70.8)	7	214	0.079		
≥ 4	91 (29.2)	7	84			
Differentiation						
G1 + G2	191 (61.2)	8	182	0.756		
G3	121 (38.8)	6	115			
Tumour morphology						
Exophytic	109 (34.9)	8	101	0.075		
Endophytic	203 (65.1)	6	197			
Deep stromal invasion						
Superficial (<2/3)	210 (67.3)	4	206	0.002*	1.307	0.743
Deep (≥2/3)	102 (32.7)	10	92			
Parametrial invasion						
Negative	296 (94.9)	8	288	5.80E-11*	14.125	0.005*
Positive	16 (5.1)	6	10			
Lymph-vascular space invasion						
Negative	197 (63.1)	7	190	0.297		
Positive	115 (36.9)	7	108			
Pelvic node status						
Negative	227 (72.8)	10	217	0.909		
Positive	85 (27.2)	4	81			
Fallopian tube invasion						
Negative	302 (96.8)	10	292	3.51E-8*	1.837	0.338
Positive	10 (3.2)	4	6			
Para-aortic node status						
Negative	305 (97.8)	13	292	0.205		
Positive	7 (2.2)	1	6			
Infiltration to vagina						
Yes	58 (18.6)	8	50	1.48E-4*	4.167	0.047*
No	254 (81.4)	6	248			
Endometrial invasion						
Negative	300 (96.2)	11	289	4.65E-4*	3.156	0.213
Positive	12 (3.8)	3	9			
Uterine corpus invasion						
Negative	252 (80.8)	5	247	1.20E-5*	5.178	0.019*
Positive	60 (19.2)	9	51			
Perineural invasion						
Negative	271 (86.9)	9	262	0.011*	0.273	0.174
Positive	41 (13.1)	5	36			

*FIGO* The International Federation of Gynecology and Obstetrics

* *p* < 0.05

**Fig. 1:**
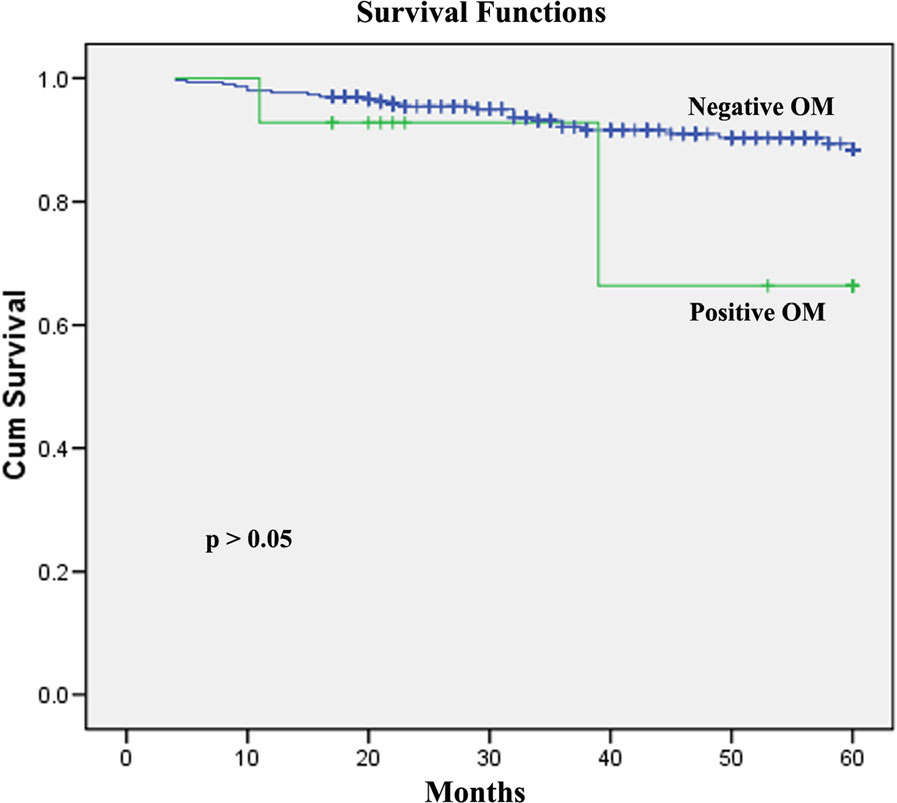
Overall survival of 14 patients positive for ovarian metastases (OM) and 298 patients negative for OM. OM had no effect on overall survival (95% CI: 0.893–9.820, *p* = 0.076)

Furthermore, multivariate analysis identified uterine corpus involvement (OR 5.178, *p* = 0.019), parametrial involvement (OR 14.125, *p* = 0.005) and vaginal infiltration (OR 4.167, *p* = 0.047) to be independently associated with OM. Meanwhile, OM had no effect on either relapse-free survival (95% confidence interval [CI]: 0.077–4.095, *p* = 0.57) or overall survival (95% CI: 0.893–9.820, *p* = 0.076) (Fig. 1).

## Discussion

ICC is still a leading cause of cancer-related deaths in women worldwide. For decades, an upward trending incidence of ADC has been reported in many countries, accounting for approximately one-quarter of all ICC cases [10]. ADC is distinguished from squamous cell carcinoma (SCC) by virtue of different human papillomavirus types, patterns of spread, prognosis and recurrence [11, 12]. Furthermore, published data has revealed that the incidence of OM in the presence of non-squamous histology is increased compared to SCC [8]; hence, bilateral oophorectomy is frequently recommended in cases of ADC.

Approximately 50% of ICC patients are premenopausal and under 45 years old [13]. Ovarian preservation has proven invaluable for improving the quality of life of these young women. According to the literature, ovarian metastatic incidence occurs more frequently among patients with ADC than in those with SCC, ranging between 1.7% and 18.9% and from 0.4% to 1.3%, respectively [6, 8, 14, 15]. Moreover, Ronnett et al. reported that in about half of ADC cases, metastases occurred post-hysterectomy [7]. Therefore, the benefits of preserving hormonal function may be offset by a potentially higher risk of recurrence in ADC. Conversely, Gubbala et al. concluded that ovarian transposition resulted in significant preservation of ovarian function with negligible risk of metastases to the transposed ovaries despite the common incidence of ovarian cysts [16]. Lyu et al. found that ovarian preservation provided oncological safety for young women with stage I cervical adenocarcinoma [17]. In the present study, the incidence of OM in ADC was 4.5% (14/312), and a significantly higher incidence of OM was observed in stage II compared to stage I cancers.

The selection of premenopausal patients who would benefit from ovarian preservation to improve the quality of life is challenging. A thorough understanding of the risk factors involved would be of great value for following a patient with ovarian preservation postoperatively [17]. The majority of cases we have examined have been diagnosed through primary radical surgery with bilateral oophorectomy. However, in the past two decades, increasing attention has been paid to OM and contributing factors. Age, FIGO stage, histology, lymph node metastases, DSI, LVSI, bulky tumor size, parametrial invasion and corpus uteri invasion have been determined to be independent risk factors for OM in ADC [8, 18–21]. In our study, we identified FIGO stage, DSI, uterine corpus invasion, endometrial invasion, parametrial involvement, PNI, fallopian tube invasion and vaginal infiltration as significantly related to OM in ADC. In our multivariate analysis, uterine corpus invasion, parametrial involvement and vaginal infiltration were independent risk or protective factors for OM in patients with ADC. The occurrence of OM had no significant relationship to either relapse-free survival or overall survival.

The routes by which ICC spreads to the ovary remain unclear. Wu et al. proposed that lymphatic spread and transtubal implantation may be possible pathways of metastases from the cervix to the ovaries [18]. Tabata et al. reported that OM may take place via hematogenous spread of cervical carcinoma [22]. Further research efforts will be required to establish the pathways involved.

## Conclusions

In conclusion, we found that cervical ADC is associated with a relatively higher risk of OM. Based on our data, we suggest that ovarian preservation may be safely performed in young patients with early FIGO stage cervical ADC without deep stromal invasion, endometrial invasion or perineural invasion, and particularly in the absence of uterine corpus invasion, parametrial involvement and infiltration to the vagina.

## Abbreviations

ADC: Adenocarcinoma
DSI: Deep stromal invasion
ICC: Invasive cervical cancer
LVSI: Lymphovascular space invasion
OM: Ovarian metastases
PNI: Perineural invasion
SCC: Squamous cell carcinoma
